# Horizontal gene transfer *via* OMVs co-carrying virulence and antimicrobial-resistant genes is a novel way for the dissemination of carbapenem-resistant hypervirulent *Klebsiella pneumoniae*

**DOI:** 10.3389/fmicb.2022.945972

**Published:** 2022-12-01

**Authors:** Ping Li, Wanying Luo, Tian-Xin Xiang, Yuhuan Jiang, Peng Liu, Dan-Dan Wei, Linping Fan, Shanshan Huang, Wenjian Liao, Yang Liu, Wei Zhang

**Affiliations:** ^1^Department of Pulmonary and Critical Care Medicine, The First Affiliated Hospital of Nanchang University, Nanchang University, Nanchang, China; ^2^Jiangxi Institute of Respiratory Disease, The First Affiliated Hospital of Nanchang University, Nanchang, China; ^3^Yichun People's Hospital, Yichun, China; ^4^Department of Infectious Diseases, The First Affiliated Hospital of Nanchang University, Nanchang University, Nanchang, China; ^5^Department of Clinical Laboratory, Medical Center of Burn Plastic and Wound Repair, The First Affiliated Hospital of Nanchang University, Nanchang University, Nanchang, China; ^6^National Regional Center for Respiratory Medicine, China-Japan Friendship Jiangxi Hospital, Nanchang, China

**Keywords:** outer membrane vesicles, carbapenem-resistant hypervirulent *Klebsiella pneumonia*e, horizontal gene transfer, virulence genes, antimicrobial-resistant genes

## Abstract

**Introduction:**

The rapidly increased isolation rate of CR-HvKP worldwide has brought great difficulties in controlling clinical infection. Moreover, it has been demonstrated that the transmission of drug-resistant genes among bacteria can be mediated by outer membrane vesicles (OMVs), which is a new way of horizontal gene transfer (HGT). The transmission of virulence genes among bacteria has also been well studied; however, it remains unclear whether virulence and drug-resistant genes can be co-transmitted simultaneously. Co-transmission of virulence and drug-resistant genes is essential for the formation and prevalence of CR-HvKP.

**Methods:**

First, we isolated OMVs from CR-HvKP by cushioned-density gradient ultracentrifugation (C-DGUC). TEM and DLS were used to examine the morphology and size of bacterial OMVs. OMV-mediated gene transfer in liquid cultures and the acquisition of the carbapenem gene and virulence gene was confirmed using colony-PCR. Antimicrobial susceptibility testing, mCIM and eCIM were conducted for the resistance of transformant. Serum killing assay, assessment of the anti-biofilm effect and galleria mellonella infection model, mucoviscosity assay, extraction and quantification of capsules were verified the virulence of transformant. Pulsed-field gel electrophoresis (PFGE), S1 nuclease-pulsed-field gel electrophoresis (S1-PFGE), Southern blotting hybridization confirmed the plasmid of transformant.

**Results:**

Firstly, OMVs were isolated from CR-HvKP NUHL30457 (K2, ST86). TEM and DLS analyses revealed the spherical morphology of the vesicles. Secondly, our study demonstrated that CR-HvKP delivered genetic material, incorporated DNA within the OMVs, and protected it from degradation by extracellular exonucleases. Thirdly, the vesicular lumen DNA was delivered to the recipient cells after determining the presence of virulence and carbapenem-resistant genes in the CR-HvKP OMVs. Importantly, S1-PFGE and Southern hybridization analysis of the 700603 transformant strain showed that the transformant contained both drug-resistant and virulence plasmids.

**Discussion:**

In the present study, we aimed to clarify the role of CRHvKP-OMVs in transmitting CR-HvKP among *K. pneumoniae*. Collectively, our findings provided valuable insights into the evolution of CR-HvKP.

## Introduction

As a human commensal and opportunistic pathogenic species, *K. pneumoniae* infection can result in acute hospital-acquired diseases (Paczosa and Mecsas, 2016). Moreover, it is also a clinical strain with extraordinarily high multidrug resistance and hypervirulence-encoding mobile genetic components (Yang et al., 2021). The uninterrupted evolution of plasmids encoding carbapenem resistance or hypervirulence leads to the emergence of new *K. pneumoniae* strains, which can be carbapenem-resistant and hypervirulent simultaneously. Therefore, much attention has been paid to the recent outbreak of carbapenem-resistant and hypervirulent *K. pneumoniae* (CR-HvKP) strains since these pathogens may lead to acute infections in comparatively healthy populations. Furthermore, such infections are hard to treat with currently available antibiotic regimens. Consequently, the prevalence of CR-hvKP has trended upward since 2010. CR-hvKP is primarily prevalent in Asia, especially China. At the same time, it has also been reported worldwide, such as in India, Singapore, Japan, Iran, the United Kingdom, Germany, the United States, Canada, Argentina, and Russia (Lan et al., 2021). In China, the prevalence of CR-hvKP infections is 0 ~ 25.8%, with large numbers of infections found in Henan and Shandong Provinces (Zhang et al., 2020). Mechanisms for the emergence of CR-hvKP can be summarized into three patterns: (i) CRKP acquiring a hypervirulent phenotype; (ii) hvKP acquiring a carbapenem-resistant phenotype; and (iii) *K. pneumoniae* acquiring both a carbapenem resistance and hypervirulence hybrid plasmid. Since mobile genetic elements transmit virulence genes and antibiotic resistance genes, the CR-hvKP strains are widely found (Zhang et al., 2016; Lee et al., 2017; Zhan et al., 2017). With their global dissemination, such organisms have the potential to be the next ‘superbug’, and public health efforts have thus emphasized the containment of CR-hvKP.

CR-HvKP outbreaks have been reported worldwide, suggesting that it does not affect the transmission potential of the host strain when acquiring resistance genes or a resistance-encoding plasmid (Zhang et al., 2016; Mohammad Ali Tabrizi et al., 2018). However, ten potentially conjugative virulence plasmids have been identified, among which only one has been demonstrated to have transferability by experiments (Yang et al., 2021). PLVPK-like virulence plasmids are not conjugative. Their transmission may need help from conjugative elements in other plasmids, while this theory has not yet been validated.

Horizontal gene transfer (HGT) is the primary type of genetic information delivery among microbes (Daubin and Szöllősi, 2016; Husnik and McCutcheon, 2018). It is well known that genetic material is exchanged between bacteria *via* HGT through three widely described pathways: transformation, conjugation, and transduction (Turnbull et al., 2016; Redondo-Salvo et al., 2020). Recent evidence has indicated that HGT processes may also be promoted by outer membrane vesicles (OMVs) (Schwechheimer and Kuehn, 2015; Kalra et al., 2016; Wang et al., 2019). OMVs are released into the extracellular environment by Gram-negative bacteria. OMVs are enriched with bioactive proteins, toxins, and virulence factors, playing a fundamental role in the bacteria-bacteria and bacteria-host interactions. Many investigations have recognized these vesicles as vectors of HGT (Schwechheimer and Kuehn, 2015). Moreover, the luminal DNA is not affected by DNases within the OMVs, thus favoring the HGT of DNA and probably conferring extra merits to OMV-releasing microbes (Yaron et al., 2000; Kalra et al., 2016). HGT has been reported in *E. coli, Acinetobacter baumannii, Acinetobacter baylyi, Porphyromonas gingivalis, P. aeruginosa,* and *Thermus thermophilus* (Yaron et al., 2000; Kalra et al., 2016; Soler and Forterre, 2020). In *Acinetobacter*, OMVs can transfer the *bla*_NDM-1_ gene (Chatterjee et al., 2017). OMVs can spread carbapenem-resistant genes in *A. baumannii* strains (Rumbo et al., 2011). In addition, OMVs are efficient vehicles for disseminating the *bla*_CTX-M-15_ gene among *Enterobacteriaceae* (Bielaszewska et al., 2020).

OMVs derived from CR-HvKP strains promote the HGT of mobile virulence elements among bacteria, resulting in drug-resistant HvKP strains (Hua et al., 2022). Furthermore, Federica et al. have modified *K. pneumoniae* OMVs through genetic engineering, which is used as carriers for transmitting drug-resistant genes in microbial communities (Dell'Annunziata et al., 2021). However, it remains unclear whether CR-HvKP-derived OMVs can simultaneously transmit virulence and drug-resistant genes, resulting in the production of new CR-HvKP strains and the further spread of CR-HvKP. Our work first showed that CR-HvKP OMVs contained both virulence and drug-resistant genes. The CR-HvKP OMVs could transfer virulence and drug-resistant genes to the ATCC *K. pneumoniae* strain to produce CR-HvKP, resulting in the phenotype with increased drug resistance and virulence. In conclusion, our data revealed the simultaneous transmission of virulence and drug-resistant genes by CR-HvKP OMVs and we clarified the potential mechanism underlying the transmission of the CR-HvKP strain.

## Materials and methods

### Bacterial strains, antibiotic susceptibility, and growth conditions

The CR-HvKP strain NUHL30457, co-producing virulence and antimicrobial-resistant genes, was extracted from the wound of a burn patient with a severe nosocomial infection and a fatal outcome at a hospital in Jiangxi Province, China, in May 2017. The species was identified using biochemical testing with the VITEK 2 compact system (bioMérieux) and 16S rRNA sequencing. The capsular serotype was determined by the nucleotide sequence of the *wzc* gene. The isolate belonged to ST86 and K2 capsular serotype. The molecular characterization of NUHL30457 has been previously reported (Liu et al., 2019), and 42 antibiotic-resistant genes were identified in the NUHL30457 genome.

Genome statistics and comparative genomic analysis revealed that as a single chromosome of 5,302,595 bp in length, the NUHL30457 genome contained four plasmids: p30457-1(215,697 bp), p30457-2 (126,149 bp), p30457-3 (89,247 bp), and p30457-4 (49,215 bp). The genome sequence of *K. pneumoniae* NUHL30457 was submitted to GenBank under accession numbers CP026586.1 (NUHL30457 chromosome) and CP026587.1-CP026590.1 (plasmids p30457-1-p30457-4). p30457-1, a virulence plasmid containing various virulence genes, including *iroBCDN*, *iucABCD*, *rmpA*, *rmpA2*, and *iutA*, was located in the *IncHI1/IncFIB* plasmid p30457-1, while no antimicrobial-resistant gene was identified. The β-lactamase-resistant genes in the NUHL30457 genome were *bla*_SHV-1_ in the chromosome, *bla*_CTX-M-65_ and *bla*_KPC-2_ in plasmid p30457-3, *bla*_NDM-1_ in plasmid p30457-4, and *bla*_CTX-M-30_ in plasmid p30457-2. The other antimicrobial-resistant genes on the chromosome were associated with MDR mechanisms, including efflux pumps, MDR tripartite systems, the MDR MAR locus, and the mdtABCD MDR cluster. MICs of the widely used antimicrobial agents were calculated using the microdilution method according to CLSI guidelines (document M100-S30). *Escherichia coli* ATCC 25922 and *K. pneumoniae* ATCC700603 were used as quality control reference strains for antimicrobial susceptibility testing.

For all experiments, CR-HvKP NUHL 30457 strain was grown in Luria-Bertani (LB) (Difco, Detroit, MI, United States) at 37°C with shaking (180 rpm) in the presence of meropenem (8 mg/l). *K. pneumoniae* ATCC700603 was used as the recipient for transformation experiments. The strain was also maintained in LB at 37°C with shaking.

### Purification of OMVs

OMVs were purified from liquid cultures of the CR-HvKP strain NUHL30457 using a previously reported method with minor modifications (Chutkan et al., 2013; Li et al., 2018). First, 2.5 ml of overnight (O/N) bacterial culture was inoculated in 250 ml of LB supplemented with 8 mg/l imipenem. The bacterial inoculum was maintained at 37°C, and the culture was orbitally shaken (180 rpm) for 8–12 h until the OD600 nm of 1.0 was achieved. The culture medium was centrifuged at 11,000 × *g* for 20 min twice at 4°C in a 50-mL centrifugal tube, and the supernatant was collected. The low-speed supernatant was transferred into a new tube, followed by centrifugation at 13,000 × g for 20 min at 4°C, and the mid-speed supernatant was harvested. The supernatants were filtered using a polyethersulfone (PES) top filter with pore sizes of 0.45 μm and 0.22 μm (Millipore, Burlington, MA, United States) to deflect remaining bacteria and cell debris. Subsequently, 23 ml cell-free supernatant was added into an ultracentrifuge tube (Beckman Coulter NO. 355618), and the medium was carefully underlaid with 2 ml of 60% iodixanol using Hamilton blunt point 4-inch needles. Then the cell-free culture supernatant was centrifuged at ultra-high speed (150,000× g, centrifuge Optima XPN-100 Beckman Coulter and rotor 70Ti) at 4°C for 1.5 h. The blunt-end needles were carefully used to collect 3 ml from the bottom of the ultracentrifuge tubes containing 2 ml iodixanol cushion and 1 ml medium, resulting in a mixture of 40% iodixanol. OptiPrep (60% iodixanol; Sigma-Aldrich) density gradient solutions with 5, 10, and 20% density gradients were prepared and distributed into discrete 5–20% density gradient layers. Subsequently, 3 ml 40% iodixanol solution containing nanoparticles was placed at the bottom of the discontinuous gradient and centrifuged at 150,000 g for 16 h at 4°C with an SW40 Ti rotor. Subsequently, phosphate-buffered saline (PBS) was used to resuspend the OMV layer, the suspension was filtered through a 0.22-μm membrane filter, and 10 μl was transferred onto an LB plate to test the bacterial growth. Bacteria-negative preparations were adopted for further analyses. The isolated OMVs were preserved at-80°C.

### Transmission electron microscopy

A transmission electron microscope (Hitachi H-7800, Japan) was used to examine the morphology and size of bacterial OMVs. Briefly, 20 μl of exosomal suspension, viral suspension, nanomaterial suspension, or other suspensions was dropped onto the copper grid with carbon film for 3–5 min. Then the excess liquid was absorbed using filter paper. Subsequently, 2% phosphotungstic acid was dropped on the copper grid to stain for 1–2 min, then the excess liquid was absorbed using filter paper, and the copper grid was dried at room temperature. The cuprum grids were examined under TEM, and images were acquired.

### Size characterization of OMVs by dynamic light scattering

Vesicle diameter size (Z-ave) and polydispersity index (PDI) analysis were performed using Zetasizer NanoZS 90 (Malvern Instruments, Worcestershire, United Kingdom). For DLS, 40 μl of OMV aliquots were gently mixed and transferred to sterile cuvettes. PBS was adopted as the dispersing solvent. All analyses were carried out at 25°C, and each purification had three replicates. All measurements were carried out 12 times per sample. DLS data were analyzed using Zetasizer software (V 7.11) supplied by Malvern Panalytical (Malvern, United Kingdom).

### Sodium dodecyl sulphate-polyacrylamide gel electrophoresis

The protein concentration of OMVs was determined using the Bradford assay (Bradford). Equal amounts of proteins were subjected to 12% SDS-PAGE. The gel was subjected to Coomassie brilliant blue staining. Protein molecular weight standards (size range 11–180 kDa, Bio-Rad).

### OMV-mediated gene transfer in liquid cultures and PCR screening

For gene transfer experiments through OMVs from CR-HvKP strain NUHL30457, OMV preparations were exposed to 50 mg/l proteinase K to digest phage coats (if present) and 2 U of DNase I (Sigma-Aldrich) to eliminate extravehicular DNA (Rumbo et al., 2011; Bielaszewska et al., 2020). The mixtures were maintained at 37°C for 20 min, followed by DNase digestion (65°C, 10 min) (Bielaszewska et al., 2020).

OMV-mediated transformation experiments were conducted as previously described (Chatterjee et al., 2017). The recipient strain *K. pneumoniae* ATCC700603 was inoculated in LB broth and grown until the OD600 nm was 0.4. Cells were diluted in cold LB at a final concentration of 107 CFU/ml. Bacterial suspensions (60 μl) were incubated with 20 μg and 50 μg of CR-HvKP OMVs statically for 4 h at 37°C, followed by incubation for 4 h under orbital shaking (180 rpm) at 37°C. Next, 10 ml of fresh LB medium was supplemented to each bacterial suspension, followed by incubation overnight under orbital shaking (180 rpm) at 37°C. Two additional experiments were carried out to further validate whether OMVs mediated the plasmid transfer: (i) free plasmid and (ii) OMVs pre-lysed with Triton X-100. On the following day, the bacterial pellet was resuspended in 1 ml of LB broth, and 10-fold dilutions were incubated overnight on LB agar (to determine total bacterial counts) or LB agar containing 2 mg/l imipenem and 5 mg/l potassium tellurite to select transformants carrying both carbapenem gene and virulence gene. The frequency of OMV-mediated transfer was determined as the number of transformants (CFU/mL on LB agar containing imipenem and potassium tellurite) in the total bacterial count (CFU/mL on LB agar). For DNA extraction, vesicles were lysed at 100°C for 10 min. The acquisition of the carbapenem gene and virulence gene was confirmed using colony-PCR. A region of the carbapenem gene and virulence gene was amplified, and the amplicon was subjected to agarose gel electrophoresis. Table 1 lists all primer sequences.

**Table 1:** Sequences of primers.

Primer	Sequence, 5′ → 3′	Gene	Product size (bp)
*KPC-Fw*	CGTCTAGTTCTGCTGTCTTG	*bla*_KPC_	798
*KPC-Rev*	CTTGTCATCCTTGTTAGGCG		
*NDM-Fw*	GGTTTGGCGATCTGGTTTTC	*bla*_NDM_	621
*NDM-Rev*	CGGAATGGCTCATCACGATC		
*rmpA-Fw*	ACGACTTTCAAGAGAAATGA	*rmpA*	434
*rmpA-Rev*	CATAGATGTCATAATCACAC		
*rmpA2-Fw*	CTTTATGTGCAATAAGGATGTT	*rmpA2*	452
*rmpA2-Rev*	CCTCCTGGAGAGTAAGCATT		
*SHV-Fw*	GCCTTTATCGGCCTTCACTCAAG	*bla*_SHV_	898
*SHV-Rev*	TTAGCGTTGCCAGTGCTCGATCA		
*aac(6′)-Ib-cr-Fw*	TTAGGCATCACTGCGTGTTC	*acc(6′)-Ib-cr*	508
*aac(6′)-Ib-cr-Rev*	TGACCTTGCGATGCTCTATG		
*CTX-M-9 group-Fw*	ATGGTGACAAAGAGAGTGCAAC	*bla*_CTX-M-9 group_	876
*CTX-M-9 group-Rev*	TTACAGCCCTTCGGCGATGATT		
*iroB-Fw*	ATCTCATCATCTACCCTCCGCTC	*iroB*	235
*iorB-Rev*	GGTTCGCCGTCGTTTTCAA		
*iutA-Fw*	ACCTGGGTTATCGAAAACGC	*iutA*	1,115
*iutA-Rev*	GATGTCATAGCCTGATTGC		
*silS-Fw*	CATAGCAAACCTTCCAGGC	*silS*	803
*silS-Rev*	ATCGGCAGAGAAATTGGC		

### Resistance assessment of transformant

#### Antimicrobial susceptibility testing

Antimicrobial susceptibility testing was conducted for transformant strain using Vitek 2 automated systems following CLSI guidelines. Results were interpreted according to the Clinical and Laboratory Standards Institute (M100-S30).

#### mCIM and eCIM

The EDTA-CIM (eCIM) and modified carbapenem inactivation method (mCIM) were conducted as previously described (Tsai et al., 2020). The mCIM test could identify the bacteria that produced all carbapenemases, while the eCIM test was performed to differentiate MBL producers from the serine carbapenemases. To perform mCIM, 1 μl loopful of the isolates was emulsified in 2 ml of tryptone soy broth (TSB). Then, one meropenem disc was immersed in the suspension at 37°C for 4 h. The MHA plate was inoculated by 0.5-McFarland standard *E. coli* ATCC25922. Meropenem disc was removed from the suspension, and excess liquid was expelled. Meropenem disc was placed on the inoculated plate and incubated at 37°C for 24 h. An inhibition zone with a diameter of 6–15 mm or the appearance of pinpoint colonies within a 16–18 mm zone around the imipenem disc indicated the presence of carbapenemase. eCIM test was carried out when the mCIM test was positive. This test was done similarly, except that 20 μl of 0.5 M EDTA was added to the TSB after the test isolate was added. Then the meropenem disc was immersed. Meropenem discs of eCIM and mCIM tests were placed on one plate and analyzed simultaneously. An increase of ≥ 5 mm in the inhibition zone for eCIM versus mCIM was considered MBL positive. In contrast, no change in zone diameter or an increase of ≤ 4 mm indicated the presence of carbapenemase.

### Virulence assessment of transformant

#### Serum killing assay

Serum bactericidal activity was determined as previously described (Liu et al., 2017). Briefly, serum was isolated from healthy controls and preserved at-80°C. An inoculum containing 106 CFU mid-log phase bacteria was reacted with 75% pooled human serum. The final mixture was maintained at 37°C, and viable counts were determined at 0, 1, 2, and 3 h. The reaction to serum killing in terms of viable counts was scored using six grades. Grade 1 refers to viable counts <10% of the inoculum after 1 and 2 h and < 0.1% after 3 h. Grade 2 refers to viable counts of 10–100% of the inoculum after 1 h and < 10% after 3 h. Grade 3 refers to viable counts that exceeded those of the inoculum after 1 h but <100% after 2 and 3 h. Grade 4 refers to viable counts >100% of the inoculum after both 1 and 2 h but <100% after 3 h. Grade 5 refers to viable counts >100% of the inoculums 1, 2, and 3 h, which was decreased during the third hour. Grade 6 refers to viable counts that exceeded those of the inoculum after 1, 2, and 3 h, and they were increased throughout this period. Each strain was tested at least three times, and the mean results were expressed as percent inoculums. The results were expressed as a percentage of inoculation, and the responses regarding viable counts were graded from 1 to 6 as previously described (Pomakova et al., 2012). A strain was categorized as serum sensitive (grade 1 or 2), intermediately sensitive (grade 3 or 4), or serum resistant (grade 5 or 6).

#### Assessment of the anti-biofilm effect

The anti-biofilm effect was assessed using a reported approach (Xu et al., 2022). Briefly, overnight cultures of OD600 nm of 0.1 were incubated with shaking (180 rpm). Cells adherent to the wells were subjected to staining using 0.1% crystal violet for 30 min. A Thermo Scientific Multiskan FC Microplate photometer was adopted to record the absorbance of each well at 540 nm. The ability of the test organisms to form biofilm was assessed using a reported approach (Extremina et al., 2011). The mean OD values of ATCC700603 and 700,603 transformants were determined, and the ODC was determined as the sum of the average OD of the negative control (ODNC) and three standard deviations of negative control (SDNC) [ODC = average ODNC + (3SDNC)]. The ability of strains to develop biofilms was divided into the following categories: OD% ODC = non biofilm producer; ODC < OD % 23ODC = weak biofilm producer; 23ODC < OD % 43ODC = moderate biofilm producer; 43ODC < OD = strong biofilm producer.

#### Galleria mellonella infection model

The Galleria mellonella infection model was adopted to assess toxicity for virulence testing (McLaughlin et al., 2014). A total of 10 larvae weighing between 250 and 350 mg (purchased from Tianjin Huiyude Biotech Company, Tianjin, China) were employed to explore the virulence level of each isolate. The insects were inoculated by injecting 1 × 10^6^ CFU per 10 μl aliquot into the hemocoel *via* the rear left pro leg, followed by a recording of the survival rate every 12 h for 3 days. All experiments were carried out three times. The recent assessment of a range of *K. pneumoniae* isolates suggests that the parameters for the Galleria model to define hypervirulence are based on a calculation of the LD50 value (Shi et al., 2018). The HvKP strain NTUH-K2044 and *K. pneumoniae* strain ATCC700603 were used as controls of high and low virulence strains, respectively. Statistical analyses were performed and visualized with GraphPad Prism 8.0.

#### Mucoviscosity assay

The mucoviscosity of the capsule of the test strains was evaluated using a sedimentation assay (Palacios et al., 2018). Briefly, cultures were maintained in LB broth overnight and then subcultured to an OD600 nm of 0.2 in fresh medium, followed by incubation at 37°C. After 6 h, cultures were normalized to an OD600 nm of 1.0 ml^−1^ and centrifuged for 5 min at 1,000 g. The supernatant was gently removed without disturbing the pellet to measure the OD600 nm. A string test was performed by stretching the bacterial colonies on sheep blood agar plates using an inoculation loop (Shon et al., 2013). Results were presented as the mean and SD of three independent experiments. The analysis was performed with Prism 8.

#### Extraction and quantification of capsules

Uronic acid was purified and quantified using a reported method (Palacios et al., 2018). Briefly, 500 μl of bacterial culture grown for 6 h and tested in the microviscosity assay was mixed with 100 μl of 1% ZWITTERGENT 3–14 detergent in 100 mM citric acid, and the mixture was incubated at 50°C for 20 min. Cells were then pelleted, and 300 μl of the supernatant was added to 1.2 ml of absolute ethanol. The mixture was incubated at 4°C for 20 min, followed by centrifugation at the maximum speed for 5 min. The pellet was resuspended in 200 μl of distilled water, to which 1.2 ml of 12.5 mM sodium tetraborate in sulfuric acid was added. The mixture was incubated at 100°C for 5 min minutes and then left on ice for 10 min. Subsequently, 20 μl of 0.15% 3-phenylphenol in 0.5% NaOH was then added. The mixture was incubated at room temperature for 5 min, followed by the absorbance measurement at 520 nm. A standard curve of glucuronic acid was generated to determine the content of glucuronic acid, which was expressed as μg OD unit−1. Data were expressed as the mean and SD of three independent experiments. Statistical analysis was carried out with Prism 8.

### Pulsed-field gel electrophoresis

Clonal relatedness was established using XbaI-PFGE (Taraka). The isolates sharing >80% similarity were defined as the same PFGE cluster (Tenover et al., 1995). The molecular marker was Salmonella serotype Braenderup strain H9812. DNA fragments were separated with a CHEF DR III apparatus (Bio-Rad; Richmond, CA, United States).

### S1 nuclease-pulsed-field gel electrophoresis

S1-PFGE was conducted to determine the plasmid location of transformants (Du et al., 2020). Briefly, total DNA was embedded in agarose gel plugs. The plugs were digested with S1 nuclease (Taraka) at 37°C for 45 min and then separated by electrophoresis. S1-PFGE was performed to confirm the acquisition of plasmids by transformant strain.

### Southern blotting hybridization

Southern blotting hybridization was performed to determine the plasmid location of *bla*_NDM-1_-carrying plasmid and virulence plasmid (Du et al., 2020). Briefly, total DNA was embedded in agarose gel plugs. The plugs were digested with S1 nuclease (Taraka) at 37°C for 30 min and then separated by electrophoresis. Labeling of the probes and hybridized with the DIG-High Prime DNA Labeling and Detection Starter Kit II according to the manufacturer’s instructions (Roche, Basle, Switzerland).

### Growth curve measurements

Overnight cultures of transformant strains were diluted until an OD600 nm of 0.05 was achieved, and the diluents were incubated at 37°C for 12 h with vigorous aeration (180 pm). Growth curves in LB medium were performed in triplicate using standard protocols.

### Plasmid stability experiments

Plasmid stability experiments were conducted as previously described (De Gelder et al., 2007). Briefly, the integrate plasmid-carrying 700,603 transformant was propagated by serial passaging for 14 days in antibiotic-free LB broth. Every 12 h, 5 μl of each culture was transferred to 5 ml of fresh LB broth. The proportion of plasmid-containing cells was determined every 24 h, and the number of colonies growing on antibiotic-free and antibiotic-containing plates was counted. Next, the integrate-carrying strain 700,603 transformants were passaged for 14 days under meropenem and potassium tellurite selection.

## Results

### Characterization of OMVs derived from CR-HvKP

**Figure 1:**
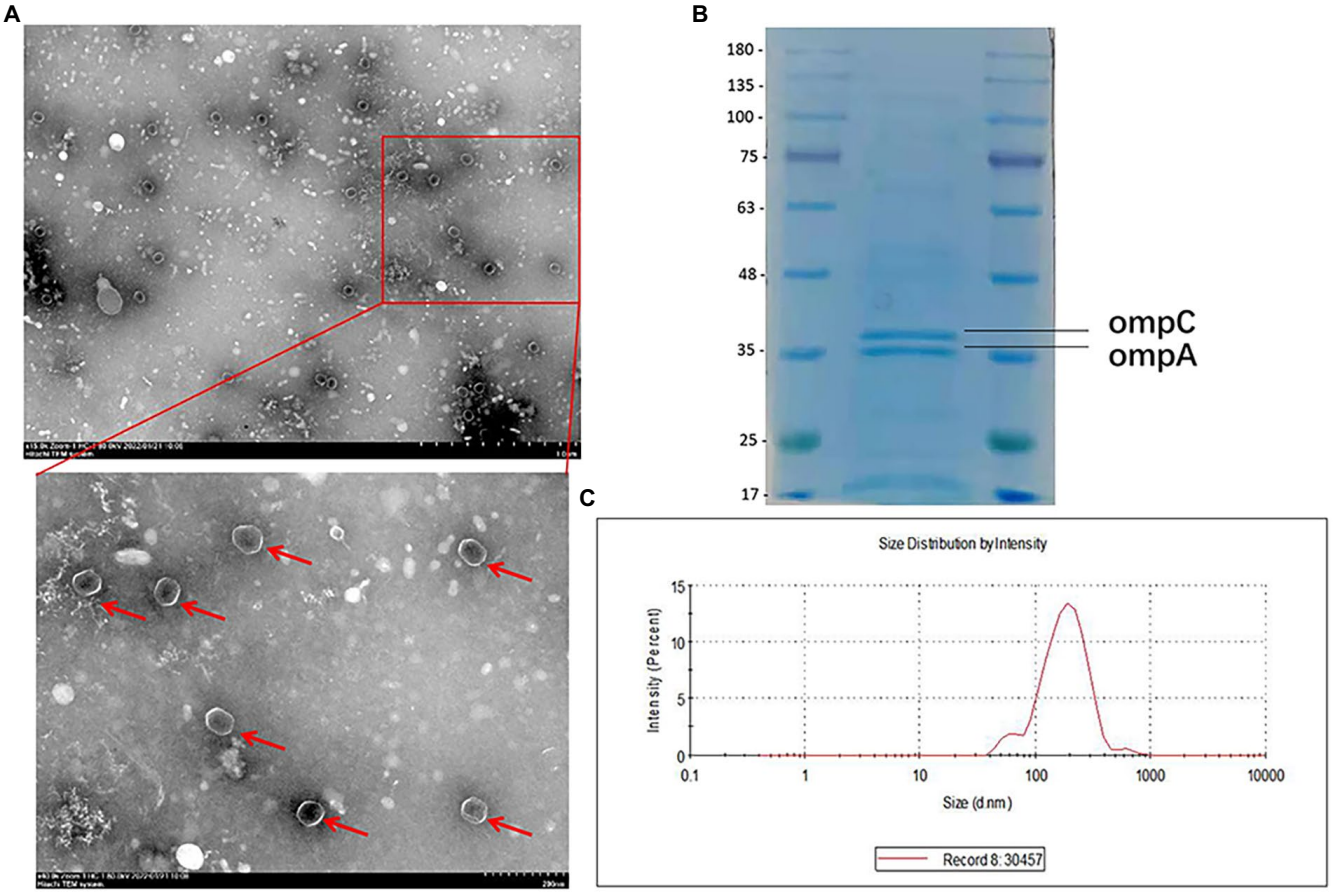
Characterization of OMVs derived from CR-HvKP. **(A)** Transmission electron microscopy (TEM) showed that CR-HvKP-derived vesicles had spherical bilayered structures. OMVS marked with red arrow. **(B)** Coomassie-stained SDS-PAGE (12%) protein profiles of CR-HvKP-derived vesicles. In the 35–48 kDa range, two major bands were detected in the OMVs. The molecular mass marker (MW) is expressed in kilodaltons (kDa). **(C)** DLS intensity-weighed distribution of OMVs derived from CRHvKP-derived vesicles.

A CR-HvKP strain (NUHL30457) (Liu et al., 2019) was grown in LB broth supplemented with 8 mg/l imipenem, and the culture supernatants were collected after 12 h of incubation. CR-HvKP OMVs were purified from culture supernatants by cushioned-density gradient ultracentrifugation (C-DGUC) and characterized concerning morphology and size. TEM showed that CR-HvKP-derived vesicles had spherical bilayered structures (Figure 1A), consistent with findings about OMVs from other Gram-negative bacteria (Dell'Annunziata et al., 2020). DLS showed that the diameters of CR-HvKP OMVs were 50–250 nm (median size of 132 nm). In contrast, we rarely found relatively large-sized vesicles with diameters >200 nm and small-sized vesicles with diameters of 50 nm (Figure 1C). This finding suggested that CR-HvKP produced and secreted OMVs into the extracellular milieu during *in vitro* culture. The protein content of OMVs was 502 ± 26 μg/ml using the Bradford assay. The protein sample of NUHL30457 OMVs was subjected to SDS-PAGE, followed by Coomassie brilliant blue staining. In the 35–48 kDa range, two major bands were detected in the OMVs (Figure 1B), which was the marker location for the ompA protein. We did not find bacterial growth on the LA plate, suggesting that the OMVs were pure. Meanwhile, no bacteria were found under the microscope.

### CR-HvKP OMVs are enriched in bacterial genetic elements

Polymerase chain reaction (PCR) results showed that carbapenemases genes and virulence genes were packaged into OMVs of NUHL30457, indicating that OMVs could contain the genetic information of strains and various virulence and drug-resistant genes (Table 2).

**Table 2:** Presence of antibiotic resistance and virulence genes in OMVs of NUHL30457 strain and 700,603 transformant.

Gene	Results of PCRs performed with the following templates					
	PK/DNase+ OMVs intact^a^	OMVs Lysed with TritonX-100 + ^b^	Purified DNA Of Omvs	700,603 transformant	Total DNA 30457	Total DNA 700603
*bla*_KPC_	+^c^	-^c^	+	+	+	−
*bla*_NDM_	+	−	+	+	+	−
*rmpA*	−	−	−	−	+	−
*rmpA2*	+	−	+	+	+	−
*bla*_SHV_	−	−	−	−	+	−
*acc(6′)-Ib*	−	−	−	−	+	−
*bla*_CTX-M-9 group_	+	−	+	+	+	−
*iroB*	+	−	+	+	+	−
*iutA*	+	−	+	+	+	−
*silS*	+	−	+	+	+	−

a, OMVs treated by PK/DNase (to remove phage coats and extravesicular DNA) and left intact. b, OMVs lysed with Triton X-100. + indicates presence of amplicon; − indicates absence of amplicon.

### CR-HvKP OMVs mediate virulence and antimicrobial-resistant gene transfer to *Klebsiella pneumoniae* ATCC 700603 strain

**Figure 2:**
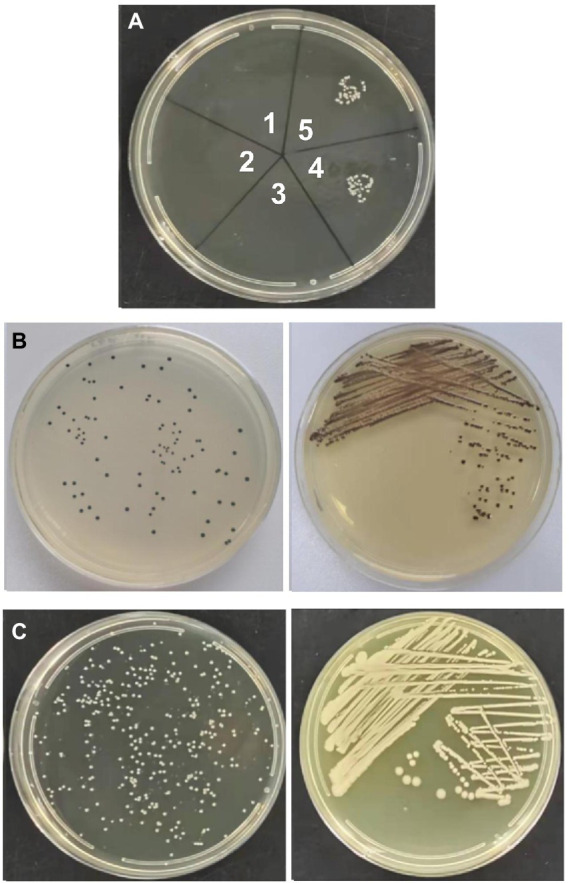
Vesicle-mediated transformation. **(A)** transformants in LB plate with 2 mg/l imipenem and 5 mg/l potassium tellurite. Note: 1. 20ug of CR-HvKP-OMVs previously lysed with Triton X-100; 2. 50ug of CR-HvKP-OMVs previously lysed with Triton X-100; 3. the free plasmid of NUHL30457; 4. 20ug of CR-HvKP-OMVs treated with DNase I and proteinase K; 5. 50ug of CR-HvKP-OMVs treated with DNase I and proteinase K. **(B)** transformants in LB plate with 5 mg/l potassium Tellurite. **(C)** transformants in LB plate with 2 mg/l Imipenem.

**Figure 3:**
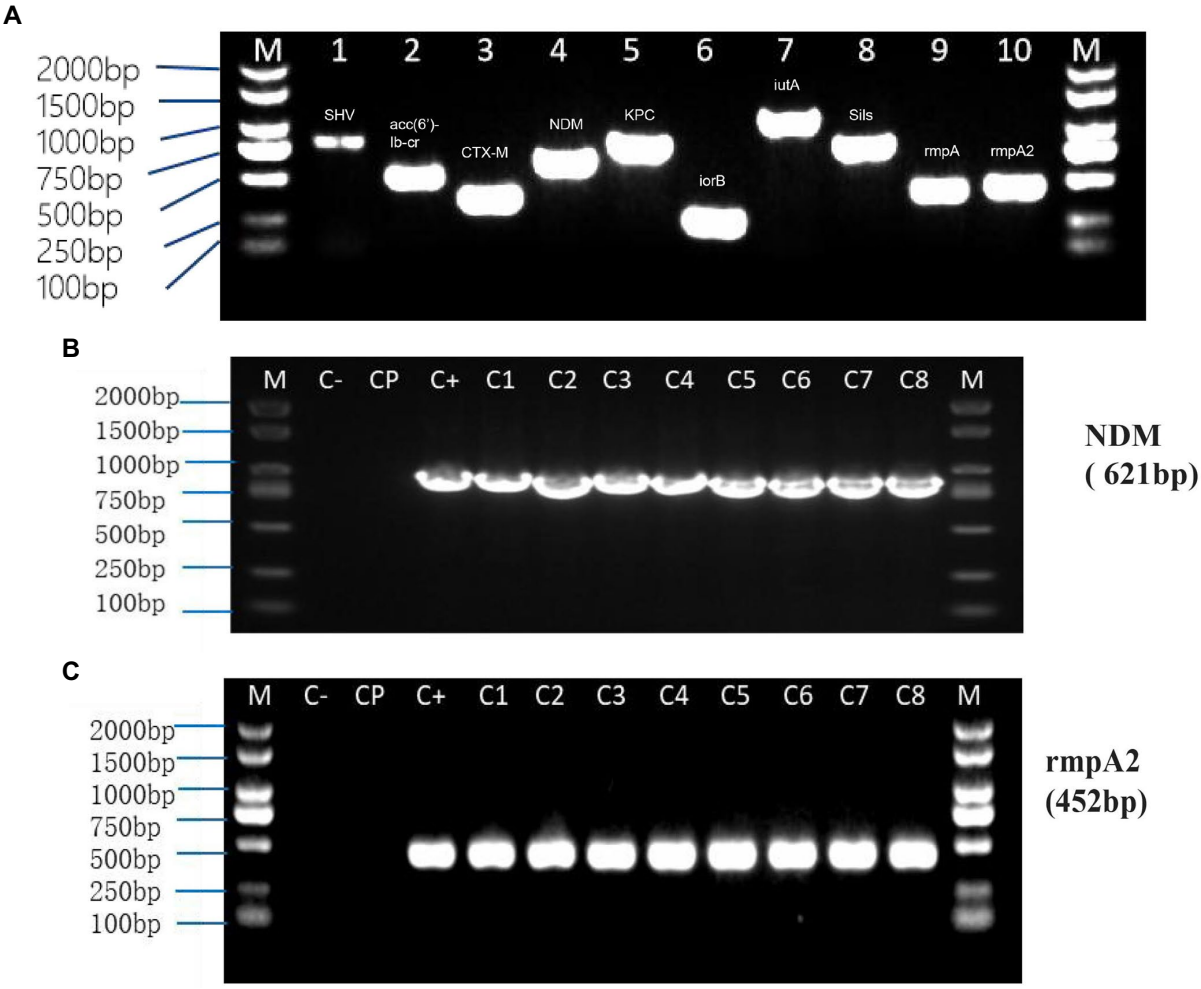
PCR from NUHL30457 omvs **(A)** and Colony-PCR from recipient cells treated with NUHL30457 omvs **(B,C)**. **(A)** NUHL30457omvs pcr, DNA gel showed PCR products with expected lengths. M: 2000 bp size marker1.*bla*_SHV_ product~898 bp 2. *acc(6*′*)-Ib-cr* product~508 bp 3.*bla*_CTX-M_ product~355 bp 4. *bla*_NDM_ product~621 bp5.*bla*_KPC_ product~798 bp 6. *iroB* product~235 bp7.*iutA* product~1,115 bp 8. *silS* product~803 bp9.*rmpA* product~434 bp 10. *rmpA2* product~452 bp **(B,C)**: Colony-PCR from recipient cells treated with NUHL30457 omvs，DNA gel showed PCR products with expected lengths. NOTE: M:2000 bp size marker Colony-PCR (C_1-8_), Control water (C−), untreated bacteria (Cp), Control water (C−), and untreated bacteria (Cp) did not show amplification.

Transformation experiments were performed by isolating OMVs from CR-HvKP NUHL30457 to determine whether CR-HvKP OMVs could transfer virulence and antimicrobial-resistant genes. The transformation experiments were successful in *K. pneumoniae* ATCC 700603 using different amounts (20 and 50 μg) of purified OMVs (Table 3; Figure 2). After 24 h, treated cells were plated on LB agar containing 2 mg/l imipenem and 5 mg/l potassium tellurite to detect the transformant’s resistance marker in the recipient bacteria. No transformants were found when the free plasmid isolated from the clinical strain CR-HvKP NUHL30457 was incubated with the ATCC 700603 strain or with different amounts (20 and 50 μg) of OMVs pre-lysed with Triton X-100. Colonies were obtained on LB agar plates at each dose of purified OMVs when ATCC700603 was transformed after incubation with OMVs purified from *K. pneumoniae* NUHL30457 (Table 3). Colony-PCR achieved the amplification of *bla*_NDM-1_ and *rmpA2* from transformants (Figure 3). One of the successful transformants was selected for further experiments.

**Table 3:** Different treatments of vesicle-mediated transformation.

Group	treatment	Amount (ug)	transformation		
			cfu/ml of transformants	Transformation frequency	Percentage of transformants
1	OMVs previously lysed with Triton X-100	20	0	0	0
2	OMVs previously lysed with Triton X-100	50	0	0	0
3	the free plasmid of 30,457	6	0	0	0
4	OMVs treated with DNase I and proteinase K	20	[(2.8 ± 0.03)x10^8^]^a^	[(1.4 ± 0.02)x10^−4^]^a^	[(1.4 ± 0.03)x10^−2^]^a^
5	OMVs treated with DNase I and proteinase K	50	[(3.79 ± 0.02)x10^8^]^a^	[(7.58 ± 0.05)x10^−4^]^a^	[(7.58 ± 0.05)x10^−2^]^a^

The data were expressed as mean ± standard deviation (SD) of three measurements; Means with lowercase letter “a” in the same column are significantly different at *p* < 0.05.

### Characterization of ATCC700603 transformant

#### CR-HvKP OMVs enhance the antimicrobial resistance-associated features of *Klebsiella pneumoniae* ATCC strains

**Figure 4:**
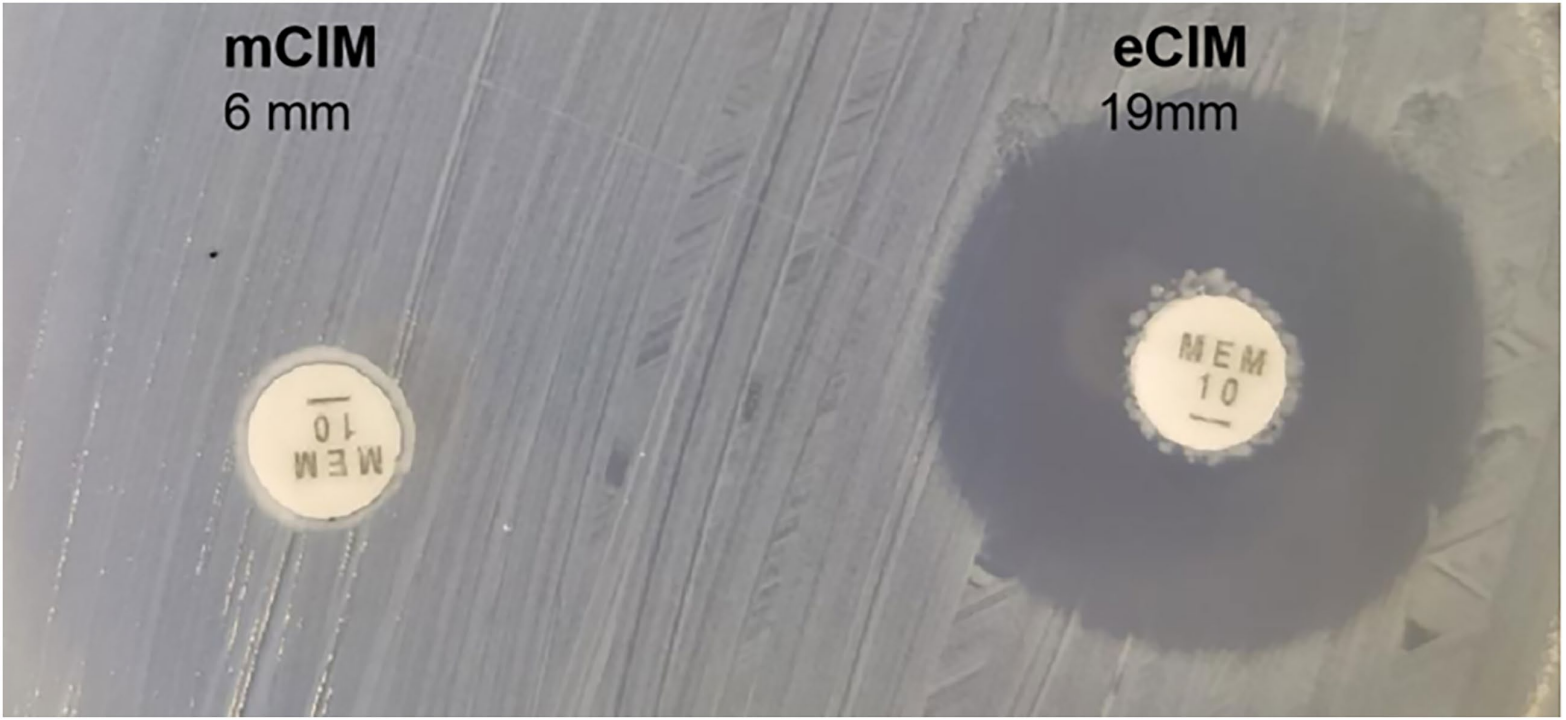
Meropenem discs of mCIM and eCIM tests of transformants. Notes:left: the inhibition zone diameter of mCIM was 6 mm; right: the inhibition zone diameter of eCIM was 19 mm.

We evaluated the phenotypic effect correlated to the genotypic resistance through antibiotic susceptibility testing. Table 4 shows the detailed antimicrobial resistance profiles. The antibiotic susceptibility test showed that the 700,603 transformant was resistant to carbapenem drugs, such as imipenem, ertapenem, and meropenem. The EDTA-CIM (eCIM) and modified carbapenem inactivation method (mCIM) were used to demonstrate that the acquired resistance was associated with the plasmid containing the carbapenemase gene. The inhibition zone diameter of mCIM was 6 mm, indicating the presence of carbapenemases. Meropenem discs of eCIM and mCIM tests were placed on one plate and analyzed simultaneously. The inhibition zone diameter of eCIM was 19 mm, which was MBL positive (Figure 4). The results showed that the transformant exhibited drug sensitivity and phenotype resistance to carbapenems.

**Table 4:** Antimicrobial resistance profiles.

Agent	NUHL30457			700,603 T			700,603	
	MIC	Interpretation		MIC	Interpretation		MIC	Interpretation
Ampicillin	≥256	R		≥256	R		≥256	R
Piperacillin/tazobactam	≥128/4	R		≥128/4	R		32/4	I
Ceftazidime	≥256	R		≥64	R		32	R
Cefepime	≥256	R		≥32	R		16	R
Aztreonam	256	R		64	R		32	R
Imipenem	≥64	R		≥64	R		≤1	S
Meropenem	≥64	R		4	R		≤1	S
Amikacin	≥256	R		≤2	S		≤ 1	S
Ciprofloxacin	≥32	R		≤0.25	S		≤0.25	S
Levofloxacin	≥32	R		1	S		≤ 1	S
Trimethoprim/sulfamethoxazole	≥4/76	R		≤2/38	S		≤2/38	S
Tigecycline	≤1	S		≤1	S		≤1	S

#### CR-HvKP OMVs improve the virulence level of *Klebsiella pneumoniae* ATCC strains

Mucoviscosity assay, uronic acid production, serum killing assay, anti-biofilm effect, and Galleria mellonella infection model were carried out on donors, receptors, and transformants to verify whether the virulence level was changed.

**Figure 5:**
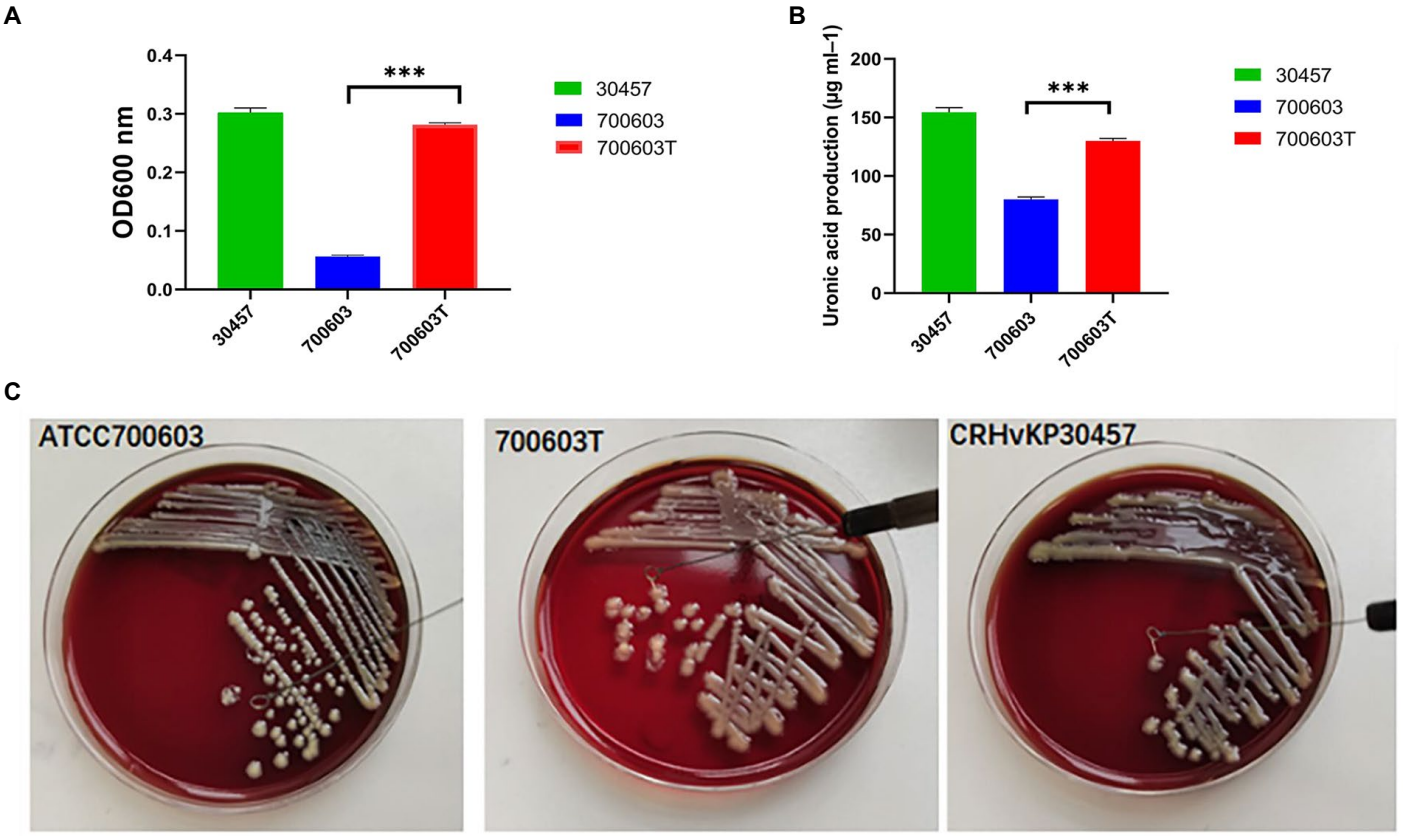
Mucoviscosity **(A)**, uronic acid production **(B)**, and string tests **(C)** of the 700,603 transformant strain.Two-way ANOVA tests were performed for statistical analysis. ***, *p* < 0.001.

**Figure 6:**
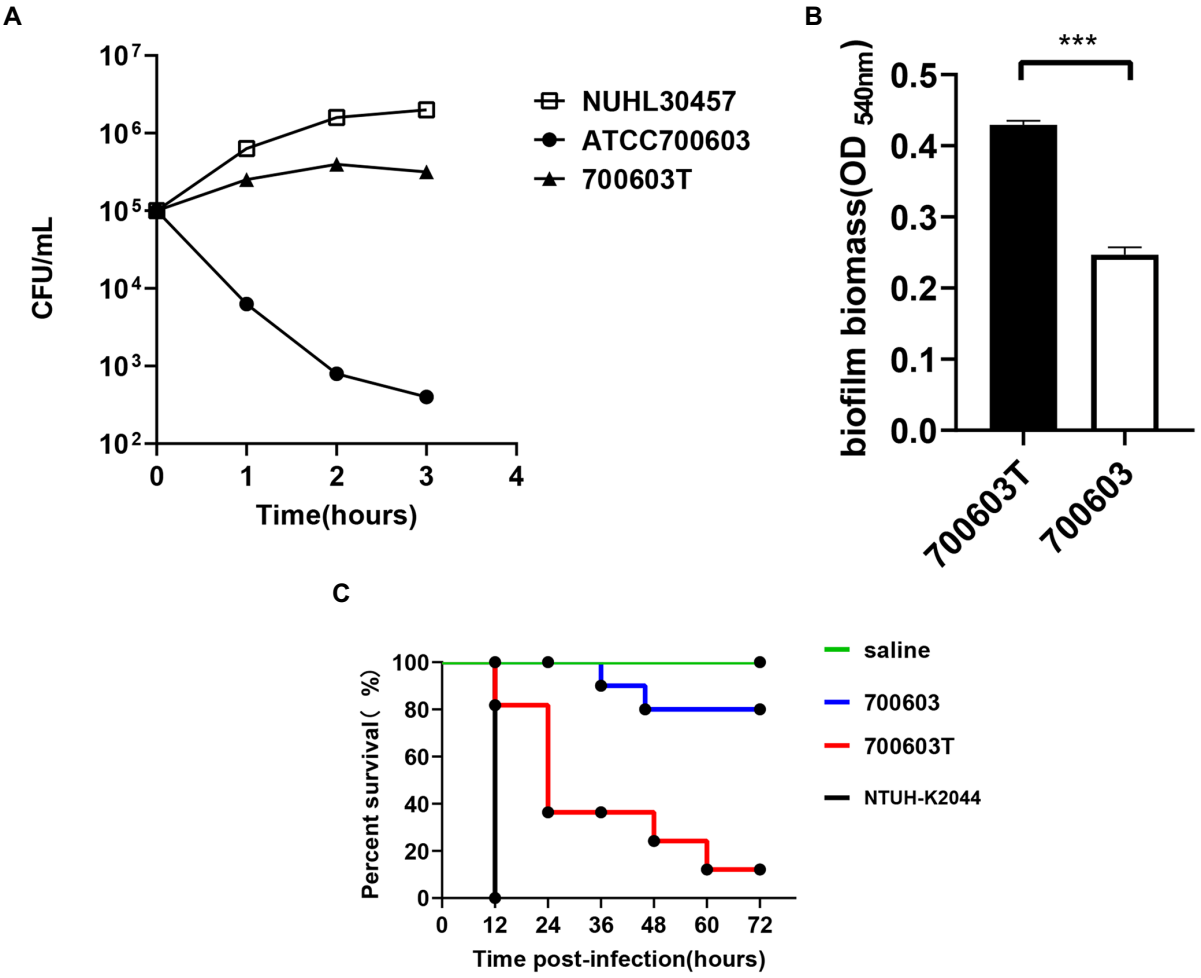
Virulence characteristics of transformants. **(A)** The serum resistance level of the 700,603 transformant strain. **(B)** The ability of the 700,603 transformant strain to form biofilm was evaluated by crystal violet assay. **(C)** The Mann–Whitney test was used for statistical analyses.***, *p* < 0.001. Virulence potential of the 700,603 transformant strain in a *Galleria mellonella* infection model.

1. To verify whether the transformant obtained the virulence gene, the expression of high viscosity phenotype was detected in the transformant. The results showed that acquisition of CR-HvKP OMVs by the ATCC 700603 strain significantly increased microviscosity and production of capsular polysaccharide (uronic acid) to a level similar to the CR-HvKP NUHL30457 strains (Figures 5A,B). In addition, string tests were favorable compared with the recipient strains, showing a markedly increased length by stretching colonies of 700,603 transformant strains (Figure 5C).
2. Serum resistance level of the CR-HvKP NUHL30457 strain, ATCC 700603 strain, and 700,603 transformant strain. The CR-HvKP NUHL30457 strain, HvKP BD2411, and 700,603 transformant strain exhibited grade 6 and grade 5 responses, respectively, whereas ATCC 700603 exhibited a grade 1 response (Figure 6A).
3. Biofilm plays a primary role in expressing the resistance and virulence phenotypes of CR-HvKP. Therefore, we investigated the ability of the 700,603 transformant strain and ATCC 700603 to form biofilm by crystal violet assay. The cut-off value (ODC) was 0.12, and the final absorbance of 700,603 T and ATCC700603 biofilm was 0.4293 ± 0.0166 and 0.2470 ± 0.0028, respectively (Figure 6B). Moreover, the two tested strains (ODtest) were considered moderate biofilm producers (2*ODC < ODtest % 4*ODC) after 24 h of incubation (Extremina et al., 2011).
4. The Galleria mellonella model showed that the 700,603 transformant strain had toxicity for virulence testing. The virulence of 700,603 T was markedly different compared with ATCC 700603 (*p* < 0.5, by log-rank test) (Figure 6C). The results showed that the transformant was a highly pathogenic phenotype.

#### PCR and S1-PFGE analysis of 700,603 transformant strain

**Figure 7:**
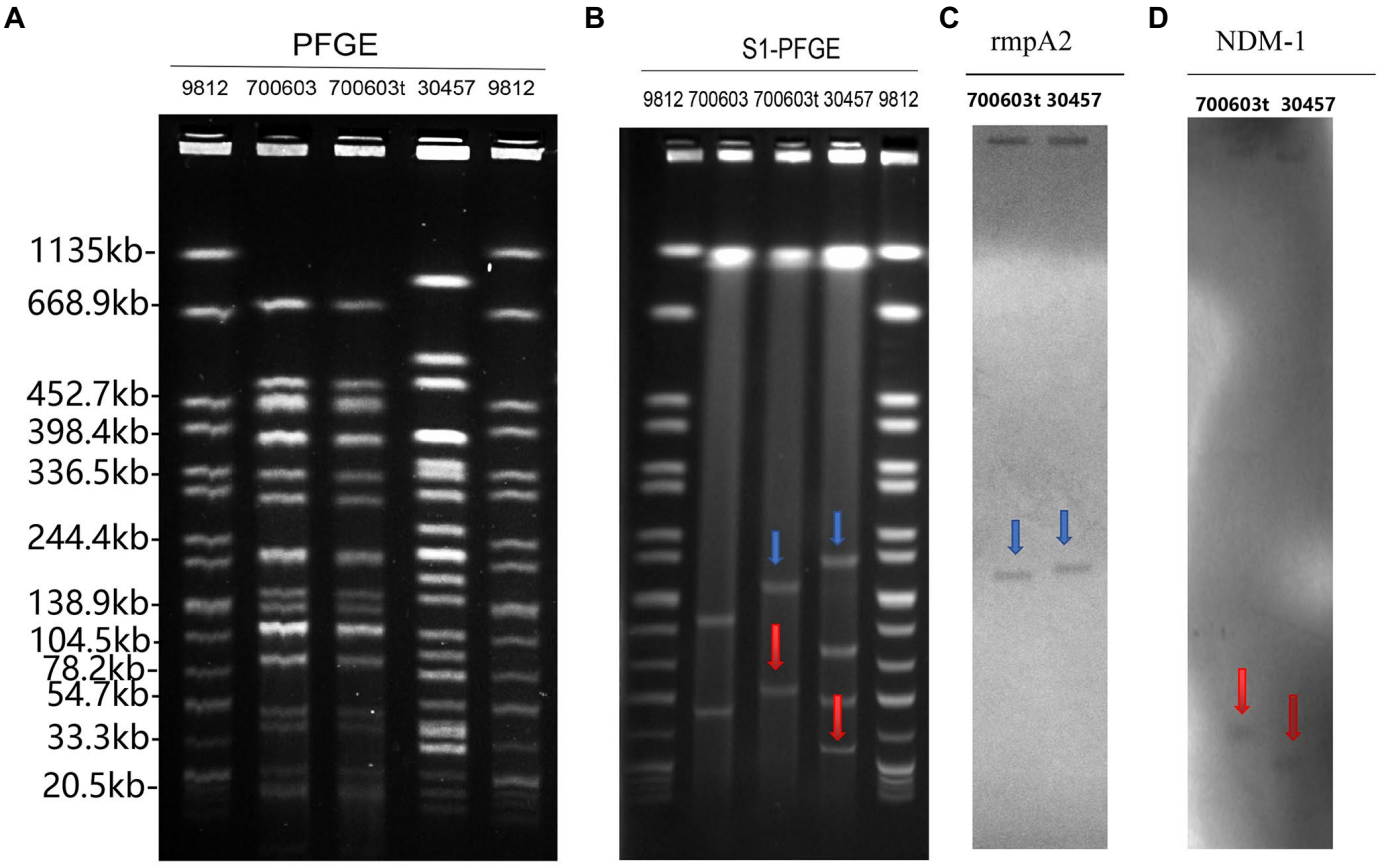
PFGE, *S1*-PFGE and Southern hybridization analysis of 700,603 transformant strain **(A)** PFGE, Clonal relatedness was established using *XbaI*-PFGE. **(B)** *S1*-PFGE: *S1* nuclease digestion of genomic DNA of *K. pneumoniae* strains was followed by PFGE. Plasmid bands are shown as linearized fragments on the gel. **(C)** Southern blot hybridization of the marker gene (rmpA2) of the virulence plasmid, marked with a blue arrow. **(D)** Southern blot hybridization of *bla*_NDM-1_ gene, marked with a red arrow. Lane 9,812, reference standard strain Salmonella serotype Braenderup H9812 restricted with *Xbal*.

PCR confirmed the presence of virulence and antimicrobial-resistant genes in 700,603 transformants. Some genes were transferred to the *K. pneumoniae* ATCC 700603 recipient cells (Table 2). To determine whether CR-HvKP OMVs transferred plasmids to transformants, we compared the plasmid profiles of the CR-HvKP NUHL30457, ATCC 700603, and 700,603 transformants. The PFGE fingerprints showed that 700,603 and 700,603 t are from the same strain and have similar homologous structures (Figure 7A). The S1-PFGE results demonstrated that 700,603 T harbored two plasmids (Figure 7B). Compared with the plasmid profiles of the ATCC700603, the positions of the two plasmids of 700,603 t were changed. The possible cause of this change was the acquisition of virulence and drug resistance genes. Southern blotting experiments confirmed that the two plasmids of the transformant contained *bla*_NDM-1_ and *rmpA2* (Figures 7C,D). Combining S1-PFGE and Southern blotting results, the plasmid of the transformant contained both drug-resistant and virulence genes, revealing that OMVs of the CR-HVKP strain could transfer virulence and drug-resistant genes.

#### Growth curve measurements and stability of plasmids in 700,603 transformant

**Figure 8:**
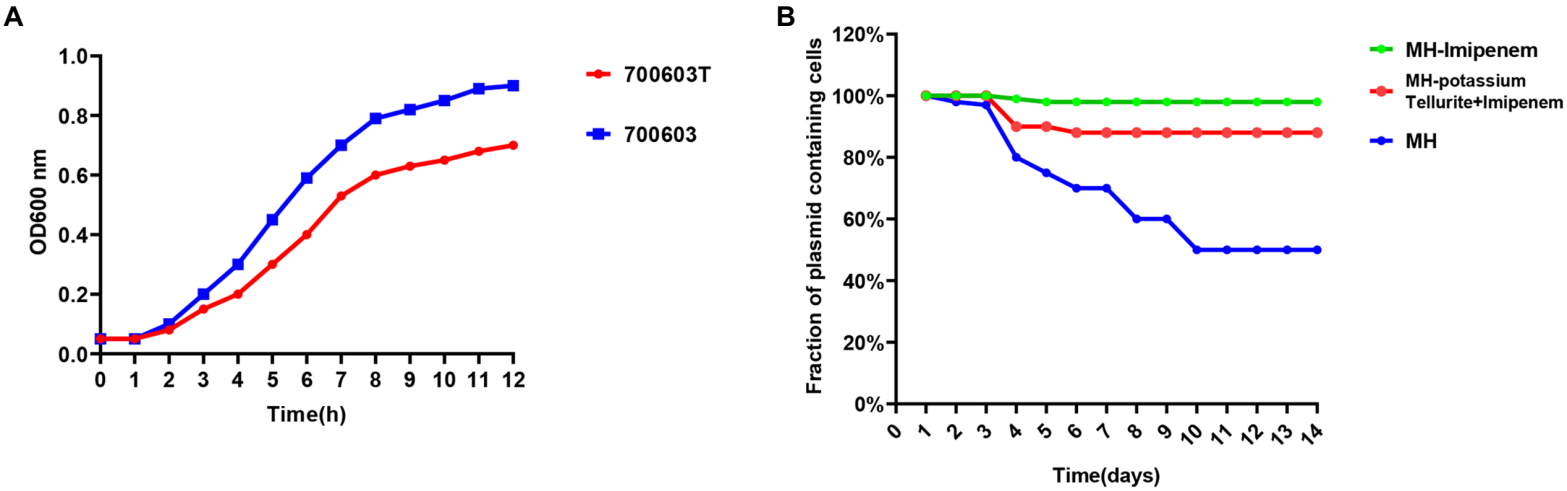
Growth curve measurements and stability of plasmids in 700,603 transformant. **(A)** Growth curves of the700603 transformant strain; **(B)** stability of plasmids in the 700,603 transformant strain in antibiotic and antibiotic-free medium.

Based on the bacterial growth curves, we found that the 700,603 transformants grew slowly compared with ATCC700603, which might be attributed to their plasmids (Figure 8A).

To evaluate the stability of plasmids in the 700,603 transformants, we propagated the plasmid harboring 700,603 in antibiotic-free LB broth for 14 days. However, the plasmid was significantly unstable because plasmid was rapidly degraded within 4 days (Figure 8B).

Given that the transformant was unlikely to persist in an antibiotic-free medium, we conducted a serial passaging experiment in LB broth containing meropenem (2 mg/l) and potassium tellurite (5 mg/l) for 14 days. After evolution under positive selection, we analyzed 32 randomly selected clones in an LB plate containing meropenem (2 mg/l) and potassium tellurite (5 mg/l) for 14 consecutive days. Under antibiotic pressure, the plasmid became stable in host bacteria (Figure 8B). However, it still had some limitations. First, the structural diversity of the plasmid under different antibiotic pressures needed to be further explored. Second, the host species used in this study were limited. Third, the underlying mechanism leading to plasmid stability under positive selection still requires further investigation.

These results supported that the transformants had drug resistance-related and highly pathogenic phenotypes. Furthermore, these findings suggested that CR-HvKP OMVs could effectively transfer virulence and drug-resistant genes to the ATCC *K. pneumoniae* strain.

## Discussion

CR-HvKP has been widely reported in Asian countries, severely threatening public health (Zhang et al., 2016; Mohammad Ali Tabrizi et al., 2018). Moreover, the rapidly increasing isolation rate of carbapenem-resistant *K. pneumoniae* worldwide (Rumbo et al., 2011) has brought great difficulties in controlling clinical infection (Schwechheimer and Kuehn, 2015). The successful persistence of carbapenem-resistant *K. pneumoniae* in clinical settings may be attributed to antimicrobial resistance and virulence. The continuous evolution of plasmids of hypervirulence or carbapenem resistance has led to the emergence of hypervirulent and carbapenem-resistant *K. pneumoniae*.

HGT is vital in promoting bacterial evolution, adaptation to environmental changes, and acquiring new metabolic capabilities (Daubin and Szöllősi, 2016). OMVs are a new mechanism to mediate the transmission of drug-resistant genes among bacteria (Husnik and McCutcheon, 2018; Soler and Forterre, 2020). Earlier reports have shown that DNA is incorporated into the lumen by OMVs and then transported to recipient cells (Rumbo et al., 2011; Kalra et al., 2016). Recent findings demonstrate that OMVs secreted by *K. pneumoniae* have been involved in HGT, allowing the spread of resistance genes in microbial communities (Wang et al., 2019). Hua et al. have demonstrated that hvKp-OMVs facilitate virulence genes transfer, allowing an increased virulence level of ESBL-producing cKp (Hua et al., 2022). A recent and detailed study has shown that OMVs derived from *K. pneumoniae* can efficiently deliver virulence plasmids into other *K. pneumoniae* strains, even carbapenem-resistant strains (Wang et al., 2022). Recently, the continuous evolution of plasmids of hypervirulence or carbapenem resistance has led to the emergence of CR-HvKP (Gu et al., 2018; Tian et al., 2021). In contrast, it remains unclear whether virulence and drug-resistant plasmids can be co-transmitted through OMVs. Our present study provided experimental evidence on the co-delivery of virulence and drug-resistant genes by CR-HVKP OMVs.

The CP-hvKP strain NUHL30457 was collected from a burn patient and exhibited three typical features of hvKP: hypermucoviscosity phenotype, serum resistance, and antiphagocytosis (Liu et al., 2019). In addition, WGS has demonstrated that four complete plasmids are obtained (Liu et al., 2019).

Firstly, OMVs were isolated from CR-HvKP NUHL30457 (K2, ST86). TEM and DLS analyses revealed the spherical morphology of the vesicles, which was consistent with earlier findings, with a similar diameter to the OMVs derived from *K. pneumoniae* ATCC 10031 (Dell'Annunziata et al., 2020).

Secondly, our study demonstrated that CR-HvKP delivered genetic material, incorporated DNA within the OMVs, and protected it from degradation by extracellular exonucleases. Our present findings and some early investigations (Rumbo et al., 2011; Bielaszewska et al., 2020; Hua et al., 2022) showed that the DNA inside OMVs was not affected by nucleases, which might be present in the environment or the host tissues. This process favors the interflow of genetic material or the horizontal transfer of DNA, thus probably conferring extra merits for gene dissemination to those OMV-releasing microorganisms.

Thirdly, the CR-HvKP NUHL30457 strain plasmids harbored blakpc-2, blaNDM-1 genes, and the virulence plasmid pLVPK carried rmpA2 genes. However, such virulence plasmids cannot self-transfer due to the absence of transfer modules (Liu et al., 2019). Therefore, we performed transformation experiments by isolating OMVs from CR-HvKP NUHL30457 to determine whether CR-HvKP OMVs could co-transfer virulence and antimicrobial-resistant plasmids.

The vesicular lumen DNA was delivered to the recipient cells after determining the presence of virulence and carbapenem-resistant genes in the CR-HvKP OMVs. After contact with OMVs, the recipient cell *K. pneumoniae* ATCC strain acquired and expressed resistance to carbapenem and potassium tellurite, proving the OMVs’ ability to carry both virulence and antimicrobial-resistant genes and promoting intraspecies HGT. The transformation did not occur when cells were incubated with the free plasmid, suggesting that vesicles could represent a physiological mechanism that exceeded environmental limits (exonuclease degradation, dilution of gene material, and long-distance transfer) and was related to the donor/recipient cell (state of competence, high vesicle-OM affinity, and correlation phylogenetics) (Dell'Annunziata et al., 2021).

Importantly, S1-PFGE and Southern hybridization analysis of the 700,603 transformant strain showed that the transformant contained both drug-resistant and virulence plasmids. Under antibiotic pressure, the plasmid became stable in the host bacteria. Furthermore, it revealed that OMVs of the CR-HvKP strain could transfer virulence and drug-resistant genes *via* plasmids. Our results supported that the transformants had drug resistance-related and highly pathogenic phenotypes. Furthermore, it suggested that CR-HvKP OMVs could simultaneously stably and effectively co-transfer virulence and drug-resistant genes to ATCC *K. pneumoniae*.

The emergence of CR-HvKP causes high mortality in clinical patients, suggesting that it is urgently necessary to clarify the molecular mechanism underlying the rapid global dissemination. However, there were several main limitations of this study. First, it was unclear what was the specific content of CR-HvKP OMVs. Second, the particular situation of its proteomics and nucleiomics was unknown. Third, it remained undetermined whether changing the structure of vesicles could block this transmission mode. These questions will be investigated in future studies.

The innovation of our article is that we selected a strain of CRHVKP as the research object. The strain and secreted-OMVs carried both virulence plasmid and drug-resistant plasmid. Through the conjugation experiment of OMVs, we screened the transformant CRHVKP on the plate of imipenem plus potassium tellurite. It was proved that our CRHVKP-OMVs transmitted both virulence plasmid and drug-resistant plasmid, transforming the sensitive KP strain into the CRHVKP strain. In summary, the formation mechanism of CRHVKP is expounded from various perspectives. Studies show that HVKP-OMVs can transfer virulence plasmids to CRKP strains to form CRHVKP (Wang et al., 2022). It can also transfer virulence and drug-resistant plasmids to sensitive KP strains from CRHVKP-OMVs to form CRHVKP. In the present study, we aimed to clarify the role of CRHvKP-OMVs in transmitting CR-HvKP among *K. pneumoniae*. Collectively, our findings provided valuable insights into the evolution of CR-HvKP.

## Data availability statement

The original contributions presented in the study are included in the article/supplementary material, further inquiries can be directed to the corresponding authors.

## Author contributions

WZ and YL designed the study. PiL and WaL performed the experiments. T-XX, YJ, LF, and PeL performed the analysis. PeL, D-DW, and WeL drafted the manuscript. All authors contributed to the article and approved the submitted version.

## Funding

Financial support was provided by the National Natural Science Foundation of China (82102411 and 81860368), the Natural Science foundation of Jiangxi Province (20202ACBL206025 and 20202ACBL206023), Jiangxi Province Double Thousand Plan scientific and technological innovation High-end Talent Project Research projects (jsxq2019201102), and The first affiliated hospital of Nanchang University Young Talents Scientific Research Breeding Fund (YFYPY202114).

## Conflict of interest

The authors declare that the research was conducted in the absence of any commercial or financial relationships that could be construed as a potential conflict of interest.

## Publisher’s note

All claims expressed in this article are solely those of the authors and do not necessarily represent those of their affiliated organizations, or those of the publisher, the editors and the reviewers. Any product that may be evaluated in this article, or claim that may be made by its manufacturer, is not guaranteed or endorsed by the publisher.
